# Impact of IL28B Genetic Variation on HCV-Induced Liver Fibrosis, Inflammation, and Steatosis: A Meta-Analysis

**DOI:** 10.1371/journal.pone.0091822

**Published:** 2014-03-17

**Authors:** Masaya Sato, Mayuko Kondo, Ryosuke Tateishi, Naoto Fujiwara, Naoya Kato, Haruhiko Yoshida, Masataka Taguri, Kazuhiko Koike

**Affiliations:** 1 Department of Gastroenterology, Graduate School of Medicine, The University of Tokyo, Bunkyo-ku, Tokyo, Japan; 2 Unit of Disease Control Genome Medicine, Institute of Medical Science, The University of Tokyo, Minato-ku, Tokyo, Japan; 3 Department of Biostatistics and Epidemiology, Yokohama City University Medical Center, Yokohama, Kanagawa, Japan; Kaohsiung Medical University Hospital, Kaohsiung Medical University, Taiwan

## Abstract

**Background & Aims:**

IL28B polymorphisms were shown to be strongly associated with the response to interferon therapy in chronic hepatitis C (CHC) and spontaneous viral clearance. However, little is known about how these polymorphisms affect the natural course of the disease. Thus, we conducted the present meta-analysis to assess the impact of IL28B polymorphisms on disease progression.

**Methods:**

A literature search was conducted using MEDLINE, EMBASE, and the Cochrane Library. Integrated odds ratios (OR) were calculated with a fixed-effects or random-effects model based on heterogeneity analyses.

**Results:**

We identified 28 studies that included 10,024 patients. The pooled results indicated that the rs12979860 genotype CC was significantly associated (vs. genotype CT/TT; OR, 1.122; 95%CI, 1.003–1.254; P = 0.044), and that the rs8099917 genotype TT tended to be (vs. genotype TG/GG; OR, 1.126; 95%CI, 0.988–1.284; P = 0.076) associated, with an increased possibility of severe fibrosis. Both rs12979860 CC (vs. CT/TT; OR, 1.288; 95%CI, 1.050–1.581; P = 0.015) and rs8099917 TT (vs. TG/GG; OR, 1.324; 95%CI, 1.110–1.579; P = 0.002) were significantly associated with a higher possibility of severe inflammation activity. Rs8099917 TT was also significantly associated with a lower possibility of severe steatosis (vs. TG/GG; OR, 0.580; 95%CI, 0.351–0.959; P = 0.034), whereas rs12979860 CC was not associated with hepatic steatosis (vs. CT/TT; OR, 1.062; 95%CI, 0.415–2.717; P = 0.901).

**Conclusions:**

IL28B polymorphisms appeared to modify the natural course of disease in patients with CHC. Disease progression seems to be promoted in patients with the rs12979860 CC and rs8099917 TT genotypes.

## Introduction

Hepatitis C virus (HCV) infection is a major cause of chronic hepatitis, liver cirrhosis, and hepatocellular carcinoma (HCC) [1]. In epidemiological studies of chronic HCV infection, age, duration of infection, alcohol consumption, coinfection with human immune deficiency virus, low CD4 count, male gender, and HCV genotype 3 have been shown to be associated with histological activity [2]–[7]. Although these factors explain part of the extreme variability seen in the progression of fibrosis among HCV-infected patients, they do not completely account for the differences. Genetic host factors have long been suspected to play a role in chronic hepatitis C (CHC) [8]–[10]. Two genome-wide association studies recently reported the susceptible loci for the progression of liver cirrhosis [11], [12].

Currently, patients with CHC are treated with a combination of peg-interferon (peg-IFN) and ribavirin [13], [14]. Telaprevir and boceprevir, two protease inhibitors, were recently approved for patients with genotype 1 in combination with peg-IFN and ribavirin. This combination has been shown to lead to substantial improvement in the sustained virologic response rate [15], [16]. Genetic variations near the interleukin 28B (IL28B) gene, encoding type III IFN-λ3, were shown to be strongly associated with the response to peg-IFN and ribavirin treatment in patients with CHC [17]–[20] and with spontaneous clearance of HCV [21]. Host immune cells produce IFN and other cytokines in response to viral infection. In response to HCV, cellular sensors detect the double-stranded RNA via retinoic acid-inducible gene-I and toll-like receptor 3 and activate a pathway to produce antiviral cytokines, including alpha and beta IFNs that trigger an antiviral response to eradicate the virus [22], [23].

Polymorphisms of genes involved in innate immunity are likely to influence the strength and nature of this defense system [24]. Moreover, IL28B polymorphisms were shown to be associated with lipid metabolism [25]. Thus, this genetic factor is thought to influence the natural course of HCV infection including liver fibrosis, inflammation activity, or steatosis. However, associations between IL28B polymorphisms and the state of background liver disease (fibrosis, inflammation activity, or steatosis) in patients with CHC remain controversial. Single studies may have limited statistical power to detect the modest effects of IL28B polymorphisms on disease progression.

Thus, we conducted the present meta-analysis to integrate the results of eligible studies and provide statistically reliable evidence of the role of IL28B polymorphisms in patients with CHC.

## Materials and Methods

### 2.1 Search strategy

An electronic search was conducted in MEDLINE, EMBASE, and the Cochrane Library for articles published prior to 30 April, 2012. Search terms included *IL28B*, *IL28*, *IL-28B*, *interleukin-28B*, *interleukin 28B*, *rs12979860*, and *rs8099917*. The search was limited to the English language.

### 2.2 Inclusion criteria

A study was included in the current analysis if it satisfied the following criteria: (1) It evaluated the associations between IL28B polymorphisms (rs12979860 or rs8099917) and liver fibrosis, inflammation activity, or steatosis. We also included studies that evaluated fibrosis or inflammation activity using the aminotransferase platelet ratio index or ALT. (2) It provided sufficient published data for estimating odds ratios (OR) with 95% confidence intervals (CIs). In case of multiple studies based on the same population, we selected the study with the largest number of participants. A study was excluded if (1) it dealt only with co-infection of HCV and human immunodeficiency virus, (2) it dealt only with patients with a specific condition such as a comorbid disease (e.g., thalassemia) or status after liver transplantation, or (3) it only used a recessive hereditary model (rs12979860 CC + CT vs. TT, or rs8099917 TT +TG vs. GG).

### 2.3 Data extraction

Two authors (M.S. and M.K.) independently screened titles and abstracts for potential eligibility and full texts for final eligibility. Disagreements were resolved by consultation with a third author (R.T.). The following information was extracted or calculated from each study: first author, year of publication, country of origin, ethnicity, sex, HCV genotype, and background liver information (fibrosis, inflammation activity, or steatosis) for each genotype. The analysis was based on the dominant model (CC vs. CT and TT in rs12979860; TT vs. TG and GG in rs8099917).

### 2.4 Definition

In some studies, mild or severe fibrosis or inflammation activity was not defined. To compare results among studies on these outcomes, we defined Ishak level F4 to F6; METAVIR, Ludwig Batts, and Inuyama level F3 to F4; and Knodell histology activity index as severe fibrosis. We also defined METAVIR A2 to A3 as severe inflammation activity.

### 2.5 Statistical analysis

The association of liver fibrosis, inflammation activity, or steatosis with the IL28B genotype in patients with CHC was assessed by summary ORs and corresponding 95% CIs. Heterogeneity among studies was examined with I^2^ statistics interpreted as the proportion of total variation contributed by between-study variation [26]. If there was no or low statistical heterogeneity among studies (I^2^<50% and P>0.05), the ORs and 95% CIs were calculated by the fixed-effects model. Otherwise, the random-effects model was adopted. When significant heterogeneity was observed, we performed a meta-regression analysis to investigate relationships between the effect of IL28B polymorphisms on liver fibrosis, inflammation activity, or steatosis; and continuous variables (proportion of patients with genotype 1 or 4 virus infection, proportion of males; and proportion of Caucasian, African-American, and Asian patients) to explore the possible reason for heterogeneity between studies [27], [28]. To check for publication bias, we used the linear regression approach described by Egger et al. [29]. All calculations were performed using Comprehensive Meta-Analysis software (Biostat, Englewood, NJ).

## Results

### 3.1 Characteristics of articles

**Figure 1:**
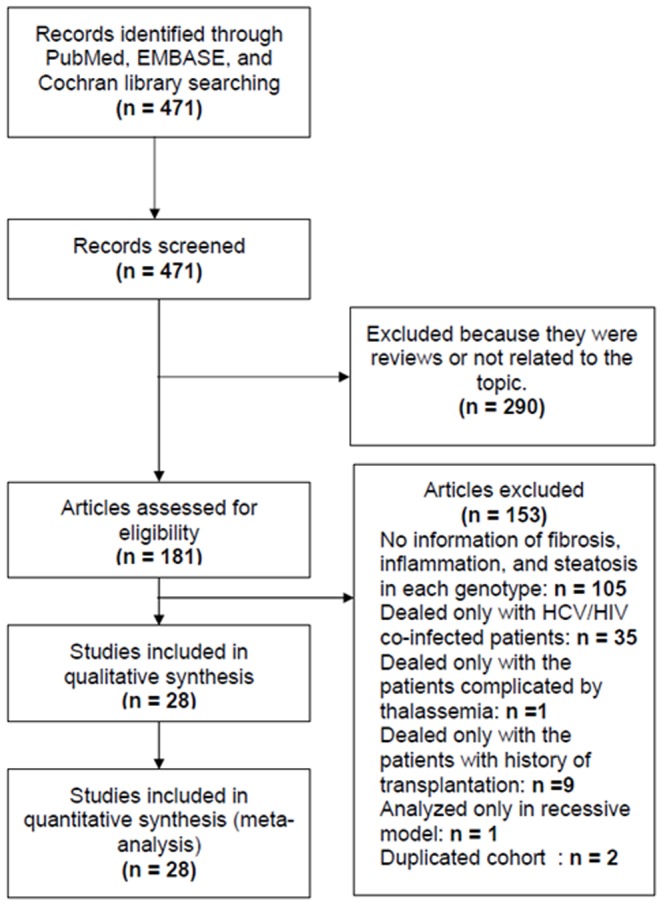
Literature search and study selection process. Twenty-eight individual studies that met all of the inclusion and exclusion criteria.

Figure 1 shows the literature search and study selection procedures. A total of 471 potentially relevant publications up to 30 April, 2012, were initially identified through MEDLINE, EMBASE, and the Cochrane Library, 443 of which were excluded because they did not meet our inclusion criteria. Therefore, 28 studies involving a total number of 10,024 patients were included in the meta-analysis. Study characteristics are shown in Table 1. There were 5616 males and 3974 females, and the sex was not reported in the remaining 434 patients (1 study). Nineteen studies (7542 patients) evaluated liver fibrosis according to rs12979860 polymorphism and 16 studies (5052 patients) according to rs8099917 polymorphism; four studies (2301 patients) evaluated inflammation activity according to rs12979860 polymorphism and eight studies (2904 patients) according to rs8099917 polymorphism; and four studies (962 patients) evaluated steatosis according to rs12979860 polymorphism and five studies (1308 patients) according to rs8099917 polymorphism.

**Table 1:** Main characteristics of all studies included in the meta-analysis.

First author (year)	Ref.	Population ethnicity, region	IL-28B SNP rsID, Allele	Outcome measure F(Fibrosis) A(Activity) S(Steatosis)	Patients*			HCV genotype	Genotype for patients rs12979860		Genotype for patients rs8099917	
					Male	Female	Total		CC	CT/TT	TT	TG/GG
Abe (2010)	[48]	Asian, Japan	rs8099917 T/G	F, A: Inuyama	212	152	364	1/2			265	99
Honda (2010)	[49]	Asian, Japan	rs8099917 T/G	F, A: Inuyama	58	33	91	1			60	31
Lotrich (2010)	[50]	Mixed (African-American/Caucasian), USA	rs12979860 C/T	F: Ishak	101	32	133	1/2	57	76		
Monte (2010)	[51]	Caucasian, Spain	rs12979860 C/T	F: Scheuer	166	117	283	1–4	129	154		
Thompson (2010)	[52]	Mixed (African-American/Caucasian/Asian/Hispanic), USA	rs12979860 C/T	F: METAVIR	986	642	1628	1	538	1090		
Bochud (2011)	[53]	Caucasian, Switzerland	rs12979860 C/T rs8099917 T/G	F: Ishak, A: ALT S: Histological finding	163	79	242	1–3	90	150	150	92
Dill MT (2011)	[54]	Caucasian, Switzerland	rs12979860 C/T rs8099917 T/G	F, A: METAVIR	30	79	109	1–4	33	96	52	57
Fabris (2011)	[44]	Caucasian, Italy	rs12979860 C/T	F: Ishak	N.A	N.A	434	1–4	133	301		
Falleti (2011)	[55]	Caucasian, Italy	rs12979860 C/T	F: Ishak	357	272	629	1–4	205	424		
Kurosaki (2011)	[56]	Asian, Japan	rs8099917 T/G	F: METAVIR S: Histological finding	250	246	496	1			269	106
Lagging (2011)	[57]	Caucasian, Sweden	rs12979860 C/T rs8099917 T/G	F: Ishak S: Histological finding	169	83	252	1–4	93	159	153	99
Lin (2011)	[58]	Asian, Taiwan	rs12979860 C/T rs8099917 T/G	F: METAVIR	123	68	191	1	171	20	170	21
Lindh (2011)-1	[59]	Mixed (Caucasian/Asian), Sweden	rs12979860 C/T rs8099917 T/G	F: Batts Ludwig	67	43	110	1	38	72	66	44
Lindh (2011)-2	[60]	Caucasian, Sweden	rs12979860 C/T	F: Ishak	204	137	341	2/3	150	191		
Marabita (2011)	[61]	Caucasian, Italy	rs12979860 C/T rs8099917 T/G	F: Ishak	129	118	247	1–4	88	159	131	116
Miyamura (2011)	[62]	Asian, Japan	rs8099917 T/G	F, A: Inuyama	37	42	79	1			53	26
Moghaddam(2011)	[63]	Caucasian, Norway	rs12979860 C/T rs8099917 T/G	F: APRI score	166	115	281	3	129	152	201	80
Rueda (2011)	[64]	Caucasian, Spain	rs12979860 C/T	F, A: Scheuer	246	177	423	1–4	83	184		
Tillman (2011)	[35]	Mixed (African-American/Caucasian/Asian), USA	rs12979860 C/T rs8099917 T/G	S: Histological finding	215	110	325	1	88	237	97	67
Yu (2011)	[65]	Asian, Taiwan	rs8099917 T/G	F: Knodell and Scheuer	264	218	482	2			315	34
Asahina (2011)	[66]	Asian, Japan	rs12979860 C/T rs8099917 T/G	F: Inuyama	28	60	88	1	54	34	54	34
Bochud (2012)	[47]	Caucasian, Switzerland	rs12979860 C/T rs8099917 T/G	F, A: METAVIR	870	657	1527	1–4	534	993	855	672
Mach (2012)	[67]	Slav: Poland	rs12979860 C/T	F: Batts Ludwig	82	60	142	1	38	104		
Miyashita (2012)	[68]	Asian, Japan	rs8099917 T/G	F, A: Desmet	88	132	220	1/2			155	63
Ohnishi (2012)	[69]	Asian, Japan	rs8099917 T/G	S: Histological finding	83	70	153	1			116	37
Rembeck (2012)	[70]	Caucasian, Sweden	rs12979860 C/T	F: Ishak	199	140	339	2/3	144	179		
Tolmane (2012)	[71]	Caucasian, Latvia	rs12979860 C/T	F: Knodell histology activity index S: Histological finding	84	58	142	1–3	41	80		
Toyoda (2012)	[72]	Asian, Japan	rs8099917 T/G	F, A: METAVIR	139	133	272	1			187	59

*Patients included in the original study.

Thus, patients without information regarding IL28B polymorphism were also included.

APRI, aminotransferase platelet ratio index.

### 3.2 Fibrosis

**Figure 2:**
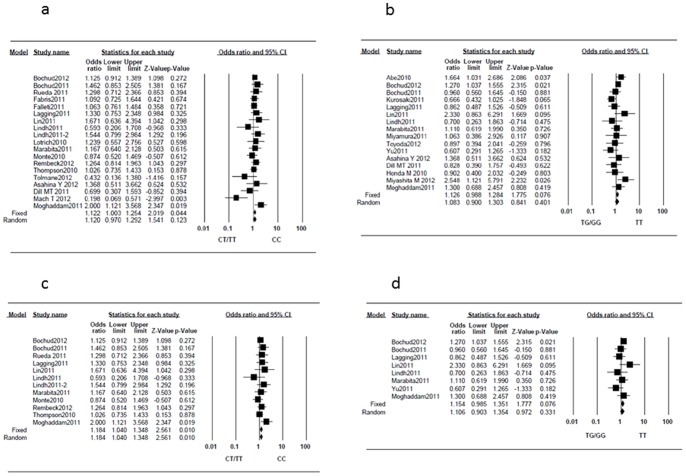
Forest plot of the IL28B genotypes and the risk of severe fibrosis. (a) rs12979860 in all patients, (b) rs8099917 in all patients, (c) rs12979860 in treatment-naïve patients, and (d) rs8099917 in treatment-naïve patients.

For rs12979860, the between-study heterogeneity was not significant (I^2^ = 25%, P = 0.147); thus, the fixed-effects model was applied. The pooled results indicated that IL28B rs12979860 genotype CC was associated with an increased possibility of severe fibrosis (OR, 1.122; 95%CI, 1.003–1.254; P = 0.044) (Fig. 2-a). For rs8099917, there was no or low heterogeneity (I^2^ = 31%, P = 0.111), and IL28B rs8099917 genotype TT tended to be associated with a higher possibility of severe fibrosis; however, the difference did not reach statistical significance (OR, 1.126; 95%CI, 0.988–1.284; P = 0.076) (Fig. 2-b). Egger's test showed no evidence for publication biases for either rs12979860 (P = 0.839) or rs8099917 (P = 0.342). When restricted to studies in which only treatment-naïve patients were included, 12 studies (5865 patients) according to rs12979860 polymorphism and eight studies (3333 patients) according to rs8099917 polymorphism were extracted. The between-study heterogeneities were not significant for rs12979860 (I^2^ = 0%, P = 0.615) and rs8099917 (I^2^ = 16%, P = 0.304). For rs12979860, fixed-effect model analyses showed a higher probability of severe fibrosis in genotype CC (OR, 1.184; 95%CI, 1.040–1.348; P = 0.010) (Fig. 2-c), and for rs8099917, genotype TT tended to be associated with a higher possibility of severe fibrosis; however, the difference was not statistically significant (OR, 1.154; 95%CI, 0.985–1.351; P = 0.076) (Fig. 2-d). Egger's test showed no evidence of publication bias (P = 0.394 for rs12979860 and P = 0.295 for rs8099917).

### 3.3 Inflammation activity

**Figure 3:**
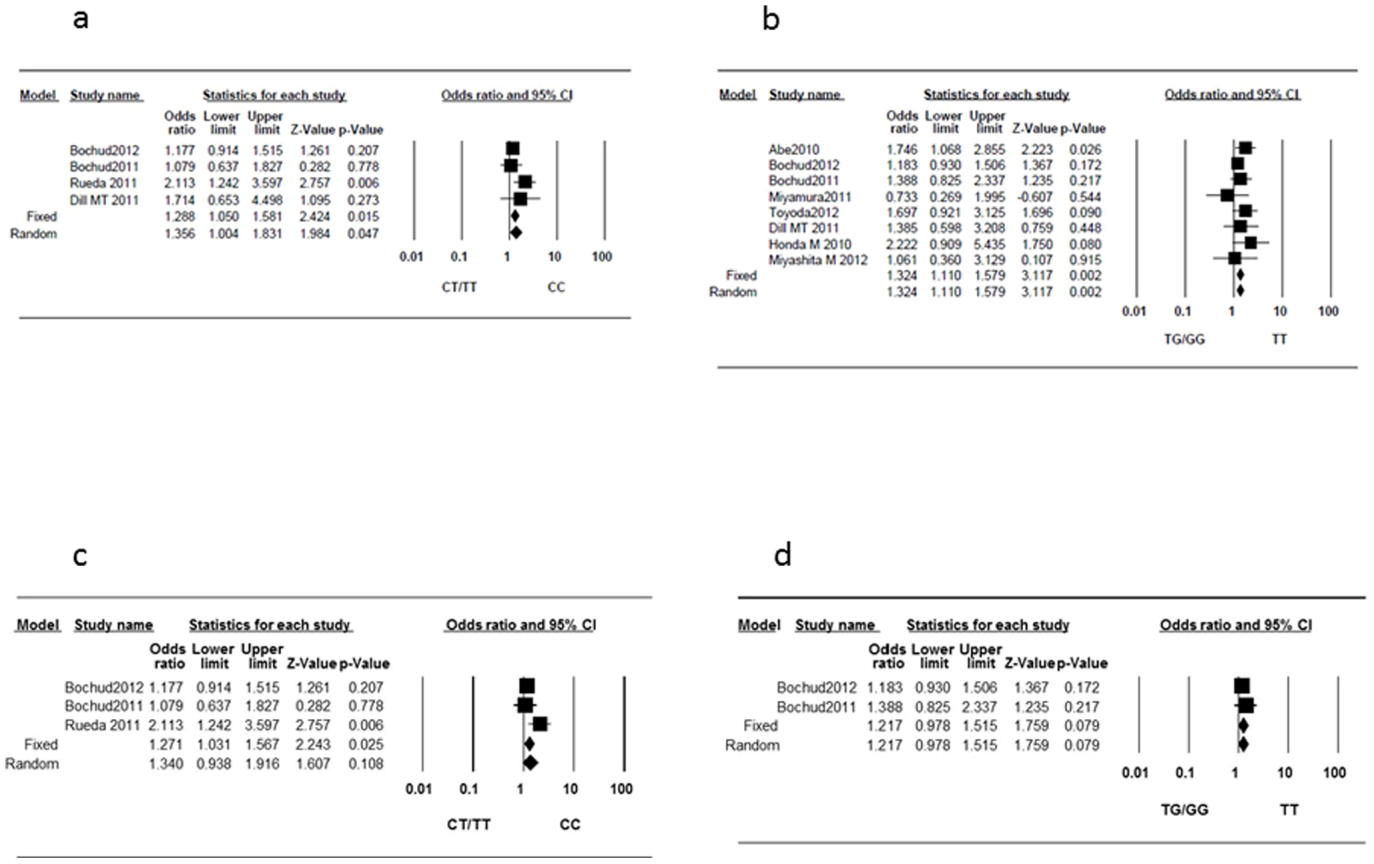
Forest plot of the IL28B genotypes and the risk of severe inflammation activity. (a) rs12979860 and (b) rs8099917. (c) rs12979860 in treatment-naïve patients, and (d) rs8099917 in treatment-naïve patients.

The between-study heterogeneity was not significant (I^2^ = 35%, P = 0.204) for rs12979860. In the fixed-effects model, the pooled results indicated that IL28B rs12979860 genotype CC was associated with a higher possibility of severe inflammation activity (OR, 1.288; 95%CI, 1.050–1.581; P = 0.015) (Fig. 3-a). For rs8099917, there was no or low heterogeneity (I^2^ = 0%, P = 0.598), and IL28B rs8099917 genotype TT was also associated with a higher possibility of severe inflammation activity (OR, 1.324; 95%CI, 1.110–1.579; P = 0.002) (Fig. 3-b). Egger's test showed no evidence of publication biases for rs12979860 (P = 0.448) and rs8099917 (P = 0.531). When restricted to studies in which only treatment-naïve patients were included, three studies (2192 patients) according to rs12979860 polymorphism and two studies (1769 patients) according to rs8099917 polymorphism were extracted. Significant heterogeneities were found for rs12979860 (I^2^ = 53%, P = 0.120); thus, the random-effect model was applied. The pooled results indicated that IL28B rs12979860 genotype was not associated with inflammatory activity (OR, 1.340; 95%CI, 0.938–1.916; P = 0.108) (Fig. 3-c). For rs8099917, the between-study heterogeneity was not significant (I^2^ = 0%, P = 0.585). In the fixed-effects model, genotype TT tended to be associated with a higher possibility of severe inflammation activity (OR, 1.217; 95%CI, 0.978–1.515; P = 0.079) (Fig. 3-d). Egger's test showed no evidence of publication bias in rs12979860 (P = 0.646). For rs8099917, Egger's test was not applicable because only 2 studies were included. We also performed a meta-regression analysis for rs12979860 because significant heterogeneities were observed. Table 2 shows the results of these meta-regression analyses. Significant correlation was observed between rs12979860 polymorphisms and the proportion of patients with genotype 1 or 4 virus (slope, 2.992±1.497; P = 0.046).

**Table 2:** Meta-regression analysis between each continuous variable among the studies (only treatment- naïve patients were included) and the effect (log odds ratio) of IL28B polymorphisms on inflammation activity.

Variables	Slope*	Standard error	P-value
Proportion of patients with genotype 1 or 4 virus, per 1% increase			
rs12979860	2.992	1.497	0.046
Proportion of male patients, per 1% increase			
rs12979860	−2.963	5.802	0.610
Proportion of Caucasian patients, per 1% increase			
rs12979860†	−	−	−
Proportion of African-American patients, per 1% increase			
rs12979860†	−	−	−
Proportion of Asian patients, per 1% increase			
rs12979860†	−	−	−

*Positive (negative) slope values indicate that the proportions of patients with the rs12979860 CC genotype with severe inflammation activity are increasing (decreasing) as the values of each contentious variable (proportions of genotype 1 or 4 virus, male, or each race) is increasing.

† We could not perform meta-regression analyses for these outcomes because only caucasian patients were included in all 3 studies included in this analysis.

### 3.4 Steatosis

**Figure 4:**
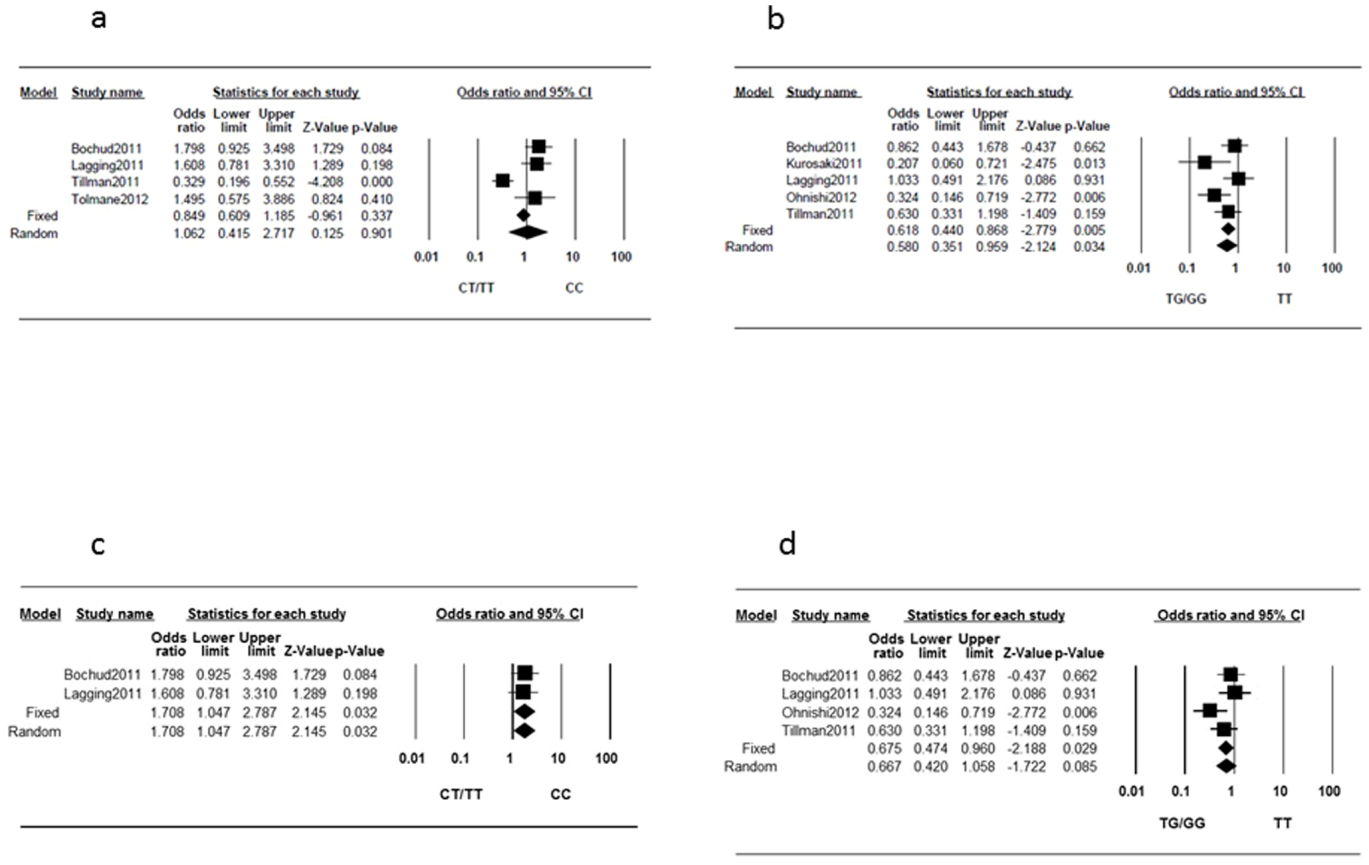
Forest plot of the IL28B genotypes and the risk of hepatic steatosis. (a) rs12979860 and (b) rs8099917. (c) rs12979860 in treatment-naïve patients, and (d) rs8099917 in treatment-naïve patients.

**Figure 5:**
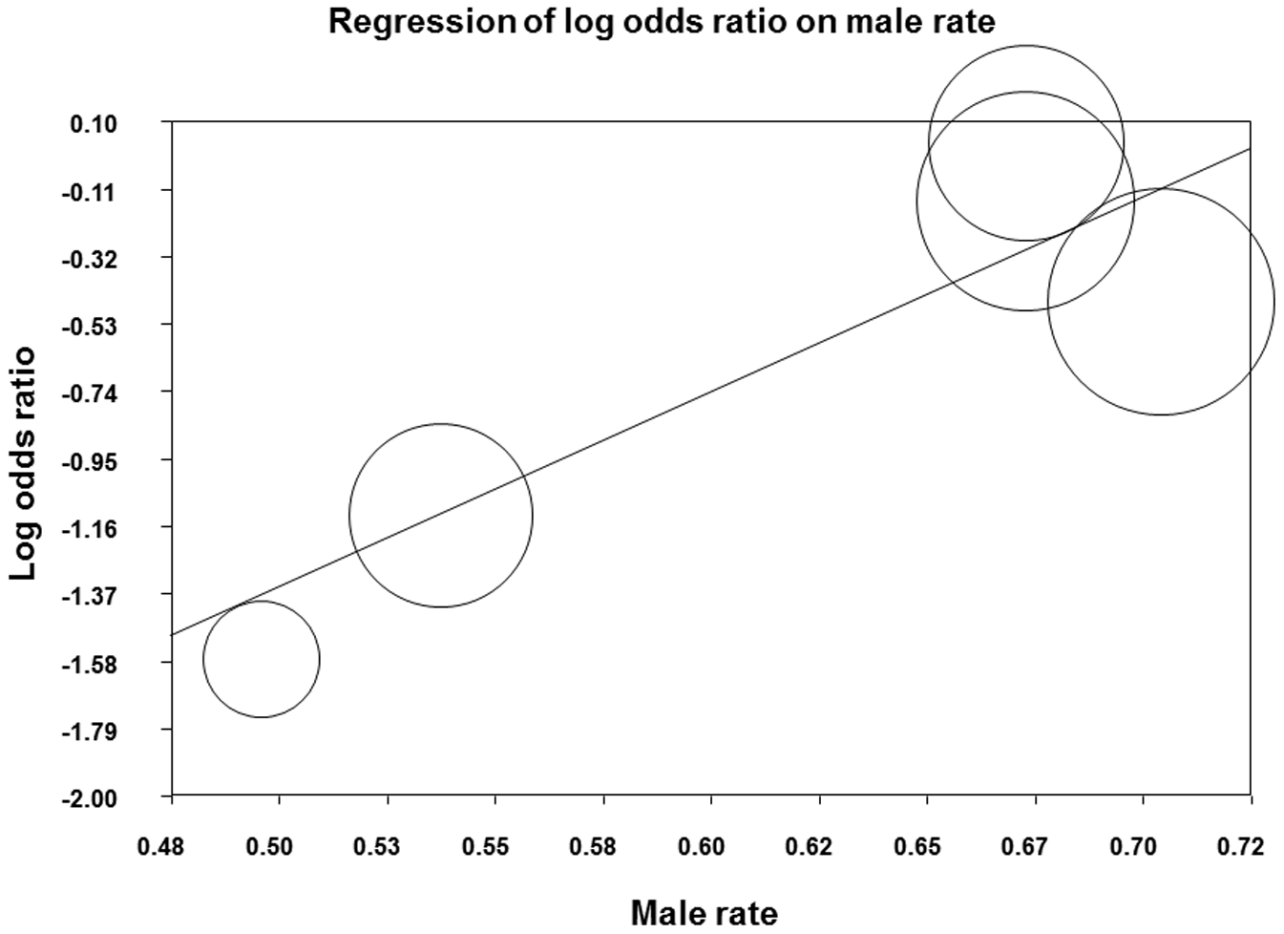
Meta-regression plot for log odds ratios in rates of patients with severe hepatic steatosis by proportion of males (%) in rs8099917.

Significant heterogeneities were found for rs12979860 (I^2^ = 86%, P<0.001) and rs8099917 (I^2^ = 52%, P = 0.082); thus, we applied the random-effects model for this outcome. The pooled results indicated that IL28B rs12979860 genotype CC was not associated with hepatic steatosis (OR, 1.062; 95%CI, 0.415–2.717, P = 0.901) (Fig. 4-a), whereas rs8099917 TT was significantly associated with a lower possibility of severe steatosis (OR, 0.580; 95%CI, 0.351–0.959; P = 0.034) (Fig. 4-b). Egger's test showed no evidence of publication biases for rs12979860 (P = 0.238) or rs8099917 (P = 0.182). We also performed a meta-regression analysis because significant heterogeneities were observed. Table 3 shows the results of these meta-regression analyses. In terms of the effect of rs12979860 on steatosis, significant correlations were observed between the proportion of patients with genotype 1 or 4 virus (slope, −4.947±1.086; P<0.001), the proportion of Caucasian patients (slope, 7.361±1.569; P<0.001), and the proportion of African-American patients (slope, −8.996±1.918; P<0.001). We also observed a significant correlation between the effect of rs8099917 polymorphism on steatosis and the proportion of male patients (slope, 6.225±2.530; P = 0.014) (Fig. 5). Finally, we observed significant correlations between rs8099917 polymorphisms and the proportion of patients with genotype 1 or 4 virus (slope, −2.704±1.277; P = 0.034), the proportion of Caucasian patients (slope, 1.168±0.422; P = 0.006), and the proportion of Asian patients (slope, −1.049±0.398; P = 0.008). When restricted to studies in which only treatment-naïve patients were included, two studies (495 patients) according to rs12979860 polymorphism and four studies (812 patients) according to rs8099917 polymorphism were extracted. The between-study heterogeneities were not significant for rs12979860 (I^2^ = 0%, P = 0.823) and rs8099917 (I^2^ = 41%, P = 0.166). For rs12979860, fixed-effect model analyses showed that rs12979860 genotype CC was significantly associated with a higher possibility of severe steatosis (OR, 1.708; 95%CI, 1.047–2.787; P = 0.032) (Fig. 4-c), whereas rs8099917 TT was significantly associated with a lower possibility of severe steatosis (OR, 0.675; 95%CI, 0.474–0.960; P = 0.026) (Fig. 4-d). Egger's test showed no evidence of publication bias in rs8099917 (P = 0.554). For rs12979860, Egger's test was not applicable because only 2 studies were included.

**Table 3:** Meta-regression analysis between each continuous variable among the studies and the effect (log odds ratio) of IL28B polymorphisms on steatosis.

Variables	Slope*	Standard error	P-value
Proportion of patients with genotype 1 or 4 virus, per 1% increase			
rs12979860	−4.947	1.086	<0.001
rs8099917	−2.704	1.277	0.034
Proportion of male patients, per 1% increase			
rs12979860	−2.899	16.577	0.861
rs8099917	6.225	2.530	0.014
Proportion of Caucasian patients, per 1% increase			
rs12979860	7.361	1.569	<0.001
rs8099917	1.168	0.422	0.006
Proportion of African-American patients, per 1% increase			
rs12979860	−8.996	1.918	<0.001
rs8099917	0.142	2.147	0.947
Proportion of Asian patients, per 1% increase			
rs12979860†	−	−	−
rs8099917	−1.049	0.398	0.008

*Positive (negative) slope values indicate that the proportions of patients with the rs12979860 CC or rs8099917 TT genotypes with severe steatosis are increasing (decreasing) as the values of each contentious variable (proportions of genotype 1 or 4 virus, male, or each race) is increasing.

† We could not perform a meta-regression analysis for this outcome because only one patient was included in the corresponding studies.

## Discussion

In the present study, we evaluated the association between IL28B polymorphisms and the background liver disease (fibrosis, inflammation activity, or steatosis) in patients with CHC. The rs12979860 CC genotype was significantly associated with a higher probability of severe fibrosis (Fig. 2-c), and the rs8099917 TT genotype tended to be associated with a higher possibility of severe fibrosis (Fig. 2-d). The accumulation of liver inflammation promotes liver fibrosis, and these polymorphisms are associated with the effect of IFN-based treatment; therefore, past treatment might alter the results. Thus, we also analyzed studies involving only patients without a history of IFN-based treatment; however, the results were not changed.

The rs12979860 CC and rs8099917 TT genotypes were also associated with a higher possibility of severe inflammation activity. Genetic variations near the IL28B gene were originally reported as strong predictors of a sustained viral response [17]–[20] or spontaneous clearance of HCV [21]. The level of IL28B gene transcripts is reportedly higher in patients homozygous for the IFN responsive allele [18], [19]. Therefore, in patients with the rs12979860 CC and rs8099917 TT genotype, IL28B production, which induces expression of interferon-stimulated genes, including some inflammatory cytokines, was thought to be increased. This may be the underlying cause of the higher inflammation activity and progressed fibrosis in patients with the IFN responsive allele. In analysis with the studies involving only patients without a history of IFN-based treatment, rs12979860 CC and rs8099917 TT genotypes were associated with higher possibility of having severe inflammation activity; however, the differences did not reach to the significant level. Only three studies according to rs12979860 polymorphism and two studies according to rs8099917 polymorphism were included when restricted to studies with only treatment-naïve patients, and may be underpowered to detect the effects of IL28B polymorphisms on inflammation activity. The further analyses with larger sample are needed to confirm this association. Additionally, meta-regression analysis showed that the effect of the rs12979860 polymorphism was influenced by viral genotype distribution. This result may imply a different influence of rs12979860 polymorphism on immune response according to viral genotype in treatment-naïve patients.

IL28B polymorphisms were also shown to be associated with lipid metabolism [25]. In the present study, the rs8099917 TT genotype was significantly associated with a lower possibility of severe steatosis. This association still remained statistically significant after we restricted to studies in which only treatment-naïve patients were included. The lower hepatic steatosis in patients with the IFN responsive allele could be explained by a more efficient export of lipids from hepatocytes. Higher interferon expression was shown to lead to suppression of lipoprotein lipase, which would result in decreased conversion of VLDL to LDL and subsequent higher steatosis [30]–[33]. The difference in IL28B expression might cause an aberration of lipid metabolism in patients with CHC. We found no significant association of rs12979860 with steatosis. And when we restricted to treatment-naïve patients, rs12979860 CC genotype was significantly associated with a higher possibility of severe steatosis. Previous studies have shown that racial differences or viral genotypes make a difference in the effects of rs12979860 and rs8099917 polymorphisms [34], [35]. This may explain the discrepancy between the effect of rs12979860 and rs8099917 on hepatic steatosis. However, only four studies (962 patients) were included in the analysis of rs12979860; or when it comes to the studies with only treatment-naïve patients, only two studies (495 patients) were extracted. Thus, we should not make any definite conclusion on this matter right now. Further studies with larger sample sizes are needed to identify their exact correlation.

According to the meta-regression analysis, the effect of rs8099917 polymorphisms on steatosis became smaller with the increase in the male proportion (Fig. 5), suggesting that a sexual dimorphism might be involved in the effect of rs8099917 polymorphisms on the liver fat content. Although the present study cannot explain the interaction between the polymorphism and sex, immune systems responding to IFN are reportedly controlled by estrogenic sex hormones [36], [37]. Differences in IL28B expression mediated by sex hormones could be a possible mechanism for the sexual dimorphism in the effect of rs8099917 polymorphisms on liver steatosis.

The rs738409 genotype within the patatin-like phospholipase domain containing 3 locus was also reported to be associated with hepatic steatosis in patients with CHC [38]–[40]. Notably, previous meta-analysis evaluating the effect of patatin-like phospholipase domain containing 3 polymorphisms on steatosis also reported a negative correlation between the male proportion and the effect of rs738409 on the liver fat content in nonalcoholic fatty liver disease [41]. Interestingly, the meta-regression analysis in the present study showed that the effect of the IL28B (rs12979860 and rs8099917) polymorphisms on steatosis was also influenced by racial and viral genotype distributions.

In the present study, we included studies that did not report the associations between IL28B genotypes and background liver diseases as study outcomes, but provided raw data that allowed us to calculate the OR for each outcome, which may have minimized potential publication bias. In fact, no publication bias was observed in the present study. The Human Genome Epidemiology Network highlighted the necessity of meta-analysis before evidence for a particular association can be regarded as strong [42]. The impact of IL28B genotypes on the disease progression found in the present meta-analysis may provide clinically important information in the follow-up of patients with CHC. The effect of IL28B polymorphisms on hepatocarcinogenesis, which is also crucial information in the HCC screening of patients with CHC, remains controversial [43]–[47]. Further analysis with larger sample sizes may be needed to elucidate the exact effect of IL28B polymorphisms on hepatocarcinogenesis.

A potential limitation of this study is inter-study variability in the outcome measure and the definition of “severe” among studies, where some discrepancies among studies exist. The studies without a pathological diagnosis, using laboratory data as surrogates, were also included. These studies may have diminished the accuracy of our research results concerning liver disease severity.

In conclusion, the present study highlighted the impact of IL28B polymorphisms on liver fibrosis, inflammation activity, and steatosis in patients with CHC. Disease progression appeared to be promoted in patients with rs12979860 CC or rs8099917 TT genotypes. The current findings may provide clinically important information in the follow-up of patients with CHC.

## Supporting Information

Checklist S1 **PRISMA 2009 Checklist.**
(DOC)
